# Left atrial myxoma complicated with multi-system embolization

**DOI:** 10.1186/s13019-017-0640-2

**Published:** 2017-09-05

**Authors:** Ren-Dan Zhang, Zhi-Huan Zeng, Jian-Yi Zheng, Tu-Di Li, Yan-Qun Zhao, Yu-Hong Liu, Yu-Si Yao

**Affiliations:** Cardiovascular Department of First Affiliated Hospital of Guangdong Pharmaceutical University, Guangzhou, Guangdong 510080 People’s Republic of China

**Keywords:** Atrial myxoma, Systemic embolism, Therapy

## Abstract

**Background:**

Atrial myxoma accounts for approximately 50% of all cardiac tumors. The majority of myxomas are located in the left atrium and present variable clinical manifestation.

**Case presentation:**

A young man was transferred to our hospital with sudden onset of resting pain, pallor and numb in right leg. An atrial mobile mass was detected by transthoracic echocardiography. Anticoagulant and antithrombotic therapy were administered, a timely surgery was performed and the mass was confirmed as a myxoma. The patient did not discharge any discomfort post-operation.

**Conclusion:**

For patients with atrial myxoma, early diagnosis is essential, anticoagulant or antithrombotic therapy and surgery have a great importance to prevent further embolism.

## Background

Primary intracardiac tumors are rare (up to 0.5% in autopsy series) and approximately 50% of them are myxomas, the majority of myxomas are located in the left atrium [1]. Patients with left atrial myxoma usually present with signs and symptoms of thromboembolic events [2]. We report an unusual case of a 25- year -old young man who presented multi-systemic embolization secondary to atrial myxoma. This case was different from other published cases mainly due to its lack of presentation or symptom prior to the embolic events. The only symptom that was relevant to the case was the atypical abdominal and right lower limb pain.

## Case presentation

A 25- year -old young man presented with sudden onset of resting pain, pallor and numb in right leg on December 16th afternoon. About 3 h after onset, he suffered from left upper abdomen cramps with nausea and voting after eating at home, every pain lasted about 10 min and occured once half an hour. So he attended to the Shenzhen baoan people’s hospital (local hospital) for medical advice on December 18th morning. Doppler ultrasonography suggested that the acute occlusions of lower extremity arteries of the right leg. Lower extremity arteries and abdominal arteries angiography confirmed the thromboembolism of the right femoral artery, right popliteal artery and the branch of ileal artery. He was diagnosed with arterial thrombosis and anticoagulant (heparin 3 mg/d, oral), thrombolytic (urokinase, 40 mg ivgtt) and antiplatelet (aspirin, 0.1 g/d, oral) were administered. About 2 h after these therapies the patient’s abdominal pain was obviously relieved but the right lower limb was still painful and numb. So he was transferred to our hospital for further treatment on December 18th afternoon.

On the physical examination, blood pressure was 140/90 mmHg in right upper limb and 110/70 mmHg in left upper limb with a heart rate of 76 beats/min, the temperature was 38.1 °C. He had normal jugular venous pressure with negative hepato-jugular reflux. On auscultation, a 2/6 grade ejection systolic murmur was heard at apex area and without a tumor plop. Abdominal examination revealed no organomegaly and abdominal tenderness. There was pallor, low skin temperature and numb in his right lower limb.

Electrocardiogram and chest radiography were normal. Hematologic laboratory values revealed leukocytosis with white blood cell 15,570/mm^3^, creatine kinase was 5583 U/L and creatine kinase isoenzyme was 41.12 U/L, erythrocyte sedimentation rate was 44 mm/h. International normalized ratio was 1.12, prothrombin time was 15.3 s, activated partial thromboplastin time was 65 s.Other laboratory findings showed no abnormalities: hemoglobin was 14.6 g/dL, platelet count was 17.3 × 10^4^/L, alanine aminotransferase was 40 IU/L, aspartate aminotransferase was 30 IU/ L, lactate dehydrogenase was 270 IU/L, low density lipoprotein cholesterol was 52 mg/dL, HbA1c was 6.0%, brain natriuretic peptide was 77.3 pg/mL, high-sensitivity reactive protein was 151.3 mg/dL, stool and urine analysis were normal.

**Fig. 1:**
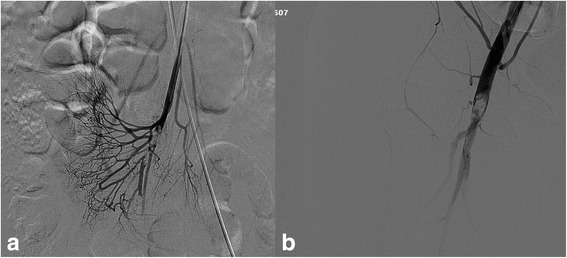
**a** Mesenteric angiography suggested superior mesenteric artery embolization. **b** Right lower limb angiography indicated that right femoral artery and right popliteal artery were occlusive, while a small amount of collateral circulation flowed to the distal arteries

**Fig. 2:**
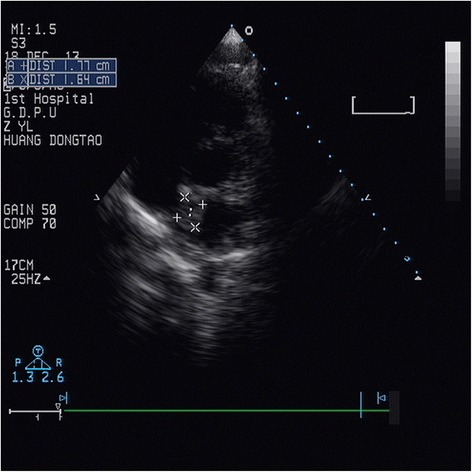
Transesophageal echocardiography demonstrating a myxoma of 1.9 × 1.2 cm rising from the left atrium

Local hospital mesenteric angiography suggested superior mesenteric artery embolization (Fig. 1a). Right lower limb angiography indicated that right femoral artery and right popliteal artery were occlusive, while a small amount of collateral circulation flowed to the distal arteries (Fig. 1b). Transthoracic echocardiography was performed and a 1.9 cm × 1.2 cm mass was seen in the left atrium which attached to interatrial septum (Fig. 2), the mass swung along with cardiac contraction. To avoid further embolization and delay in treatment, the patient underwent thoracotomy of tumor resection surgery: Our patient underwent median sternotomy under general anesthesia. Cardiopulmonary bypass (CPB) was established with conventional mild hypothermia (32.0 °C). During an anoxic arrest for 30 min with single aortic cross-clamping, the tumor and about 0.8 × 0.6 cm of the interatrial septum were excised through an oblique and longitudinal atriotomy. Our patient remained hemodynamically stable and then an embolectomy was perfomed: the embolectomy was conducted through the right femoral arteries using Fogarty catheters: two dark red foreign substance were removed from the superficial femoral artery.

**Fig. 3:**
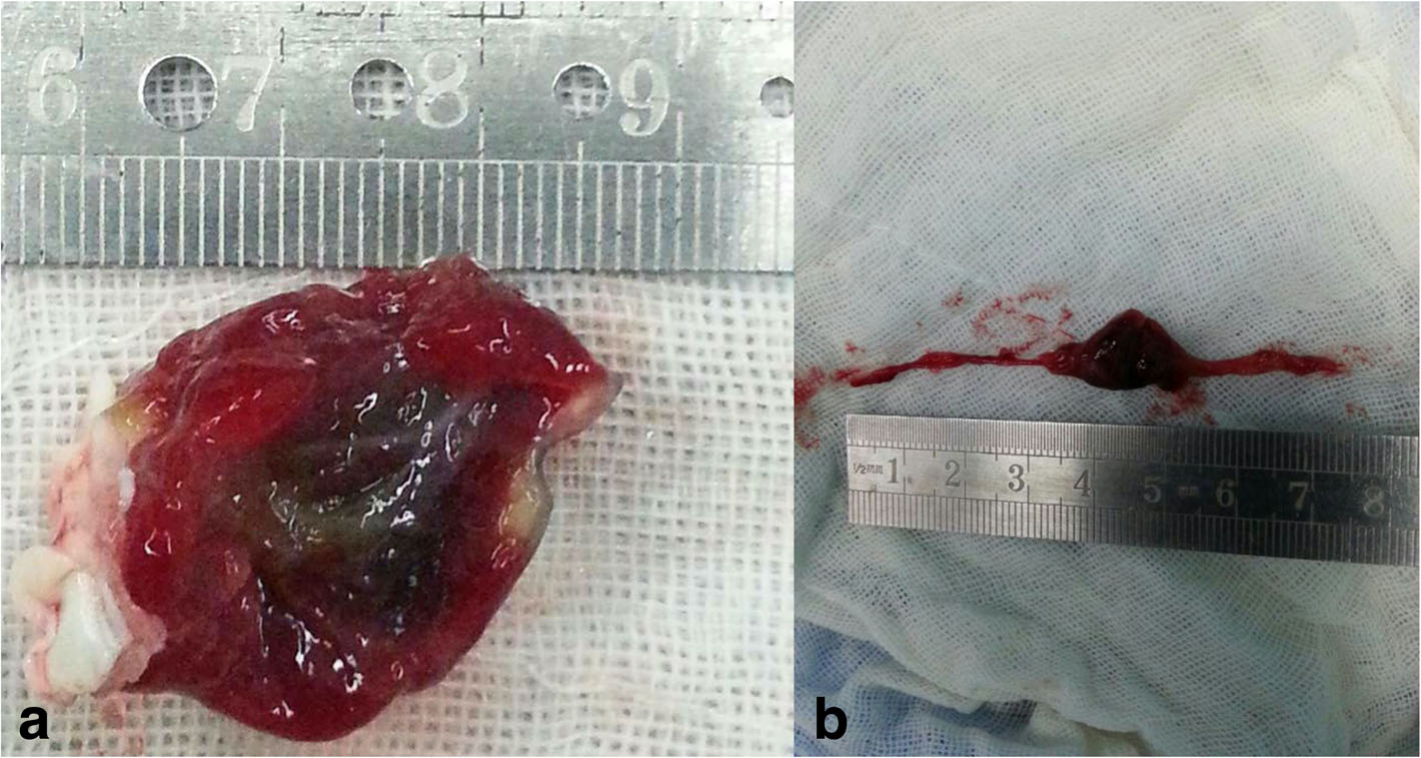
**a** The tumor was 3.0 × 2.0 cm in size, irregular and dark brown gelatinous appearance, no thrombus was found on the surface, about 0.8 × 1.0 cm of the mass was partly torn. **b** The embolus removed from femoral artery, with the same size of the collar

**Fig. 4:**
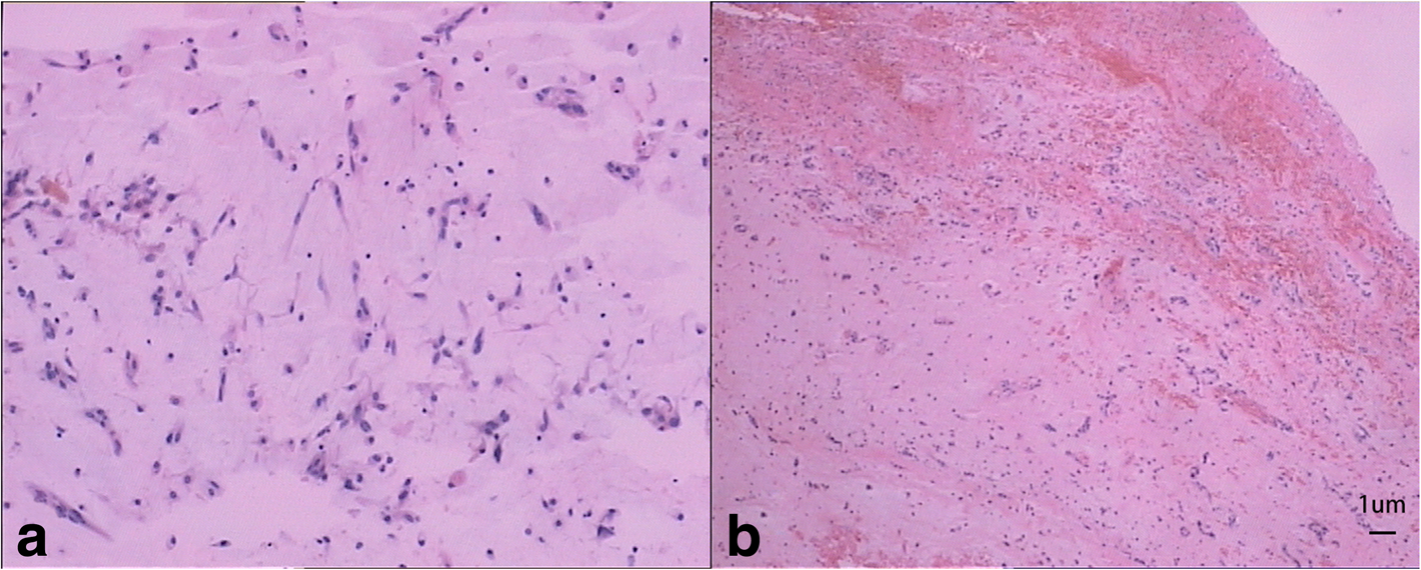
**a** Prominent spindle/ovoid/stellate cells could be seen around the blood vessels, with blue-gray mucopolysaccharide background. **b** Sections of specimens suggested the tumor was rupture with bleeding

The size of the tumor was 3.0 × 2.0 cm and the weight was 110 g in macroscopically, the appearance was irregular and dark brown gelatinous, about 0.8 × 1.0 cm of the mass was partly torn and there was no thrombus on the surface (Fig. 3a). The emboli removed from his femoral artery, the bigger size was 2.0 × 1.0 cm (Fig. 3b). Microscopically, it is showed that prominent spindle/ovoid/stellate cells around blood vessels (Fig. 4a). Sections under microscopic examination showed tissue with extensive area of hemorrhage (Fig. 4b). Therefore, the tumor was confirmed as a myxoma and it had identical microscopic finding with the embolus. The patient was discharged from hospital with recovery twenty days after the operation. He was in good fit three years after the initial attack, without recurrence of cardiac myxoma.

## Discussion and conclusion

Cardiac myxoma is a tumor of mesenchymal origin accounting for half of all primary cardiac neoplasms, right atrial myxomas are uncommon which being three to four times less frequent than those located in the left atrium [3, 4]. Cardiac myxomas develop in the left atrium and most of which are from the atrial septum near the fossa ovalis. Myxomas can present at any age group but occurs more often between the 3rd and 6th decades of life [5]. We used “embolization and atrial myxoma” as the search conditions, the results was shown in Table 1 which was all cases of atrial myxoma reported during 2012–2017. The incidence of male and female is 18 (39.1%), 28 (60.9%) respectively and most of the age of patients is forty to sixty. Furthermore, myxoma which located in left atrium accounts for the most of occurrence. As for our case, a young male with left atrial myxoma was observed, it suggests us although in young patients, the possibility of atrial myxoma should be also taken into account.

**Table 1:** A list of the reports published during 2012–2017

Characteristics	n (%)
Sex	
Female	28 (60.9)
Male	18 (39.1)
Age (years)	
< 40	12 (26.1)
40–60	18 (39.1)
60–70	5 (10.9)
> 70	11 (23.9)
Location	
LA	31 (67.4)
RA	11 (24.0)
LV	2 (4.3)
RA and RV	2 (4.3)
Organs of embolization	
Cerebral artery	10 (21.7)
Pulmonary artery	14 (30.5)
Coronary artery	4 (8.8)
Muti-system	4 (8.8)
Others (Peripheral Vessel, etc.)	12 (26.2)
Treatments	
Surgery	40 (46.9)
Thrombolytic and surgery	6 (13.1)
Total	46

Patients with myxomas usually have nonspecific symptoms, since the ability to embolize it can cause fatal complications. The probable reason for the spontaneously myoxma rupture is the tumor generally has a soft, gelatinous consistency; and squeezed by the heart for a long time which surface is usually covered with thrombosis, also the large size of a tumor, irregular surface, and atypical location may lead to a higher risk of cardiac myxoma rupture [6]. As the result listed in Table 1, ischemic stroke symptoms, chest pain and dyspnea, cardiac infarction events and constitutional symptoms were observed in 10 (21.7%), 14 (30.5%), 4 (8.8%) and 16 (35%) patients, respectively. And combined Figs. 3a and 4b, which give a good explanation of the source of the outer emboli that result in the patient’s abdominal pain and lower limb pain in our case.

An auscultation characteristic of myxoma is the “tumor plop”, it is a characteristic low-pitched sound that may be audible during early or mid-diastole which is thought to result from the tumor abruptly stopping as it strikes the ventricular wall. “Tumor plop” is a feature of the atrial myxoma, however, it only occurs in 15% cases [7] and our case without a tumor plop. Except this, transthoracic echocardiography (TTE), transesophageal echocardiography (TEE), computed tomography scan (CT) and cardiac magnetic resonance (CMR) imaging are the techniques being used for diagnosis [8]. TTE and TEE provided a good visualization of the mass with a sensitivity of 95% and 100% respectively [9]. Though echocardiography considered as the first-line imaging modality, cardiac CT has become an increasingly utilized tool for the evaluation of cardiac masses, which offers high isotropic spatial and temporal resolution, fast acquisition times, and multiplanar image reconstruction capabilities [10].CMR could also strengthen the diagnostic accuracy by providing superior soft tissue contrast which distinctly detects the internal details of myxomas, such as rupture and hemorrhage. The patient in our case immediately performed TTE when transferred to our hospital, combined with symptoms, the diagnosis is clear. In patients with atrial myxoma, TTE or TEE is still the most important means to diagnosis.

There is still no consensus whether preoperative angiography should be performed or made in the presence of ischemic symptoms [11]. On account of myxoma can be easily diagnosed by echocardiography, some authors proposed to resect the myxoma as soon as the diagnosis is made [12]. Others consider coronary angiography as necessary test only for patients with chest pain or over forty years of age [13]. In the presence of risk factors for coronary heart disease, additional coronary angiography or CT coronary angiography should be performed so that any coronary stenosis can be avoided during cardiac tumor surgery. Angiography is also sometimes helpful to determine the extent of highly vascular tumors, e.g. angiosarcoma [6]. Furthermore, embolization of myxoma fragments could occur in the coronary system [14]. It is crucial to detect this counterpart association of coronary artery disease (CAD) with cardiac tumor. Myxomas related emboli in the coronary vasculature are uncommon, but when present, they may cause fatal myocardial infarctions (MI) [15]. A previous review documented that inferior myocardial infarctions have been seen in 63.6%,anterior in 22.7%, and posterior in 9.1% secondary to myxomas embolization [16]. Patients with myocardial infarction and concomitant myxomas often constitute a complex puzzle to put together and all clinical and imaging modalities must be used to plan and implement the best therapeutic strategy [17]. And it is suggest that investigation of the coronary artery tree is remarkable in all instances before performing cardiac surgery even in the case of young population [18]. In our case, lower extremity arteriography helped clear the diagnosis of peripheral embolization, and determine the site of embolization, which was useful for embolectomy. In order to exclude CAD and left atrial embolism, we suggest do coronary angiography, unfortunately, the patient’s family does not agree.

Surgical excision of cardiac myxoma shows curative with few recurrences at follow-up observation, which carries a low operative risk and proves the primary treatment. After diagnosis, surgery should be done timely in order to prevent complications such as embolic events or obstruction of the mitral orifice. As mentioned above, anticoagulant and antithrombotic therapy turned out to be good result with our patient. As is the case reported a 54-year-old female whose clinical manifestation of atrial myxoma was an ischemic stroke [19], intravenous heparin relieved her symptom. It is considered that no matter neurologic or peripheral embolism, anticoagulation and thrombolytic therapy are practicable. Easement of abdominal pain is the result of timely thrombolytic therapy.

In conclusion, this case indicates that: 1. On a left atrial mass, differential diagnosis between thrombus and myxoma may be confused, angiography is helpful for the diagnosis, the status of TTE or TEE in the diagnosis of myxoma is irreplaceable. 2. When differential diagnosis is controversial and there is the possibility of thrombosis, a trial of anticoagulation or thrombolytic therapy should be advised, surgical treatment is still the most important means. 3. For young patients with no significant risk factors for arteriosclerosis, when multiple embolization occurs, it is need to take into account the possibility of atrial myxoma.

## Abbreviations

CMR: Cardiac magnetic resonance
CT: Computed tomography scan
LA: Left atrial
TEE: Transesophageal echocardiography
TTE: Transthoracic echocardiography
